# Self-Medicating Social Pain? Bullying, Suicidality, and Opioid-Specific Risk in US Adolescents

**DOI:** 10.1542/pedsos.2026-001614

**Published:** 2026-07-24

**Authors:** Mario Morales, Maeve E. Wallace, Beth E. Meyerson, Jennifer S. De La Rosa

**Affiliations:** 1 Health Promotion Sciences, Mel and Enid Zuckerman College of Public Health, University of Arizona, Tucson, Arizona; 2 Comprehensive Center for Pain & Addiction, University of Arizona, Tucson, Arizona; 3 Harm Reduction Research Lab, College of Nursing, University of Arizona, Tucson, Arizona; 4 Department of Family and Community Medicine, College of Medicine-Tucson, University of Arizona, Tucson, Arizona; 5 Department of Pharmacology, College of Medicine-Tucson, University of Arizona, Tucson, Arizona

## Abstract

**OBJECTIVE:**

Many adversities predict adolescent substance use, but it is unknown which produce opioid-specific risk vs general substance use liability. We tested whether bullying, suicidality, polysubstance use, risk behavior, sexual victimization, household adversity, and community safety adversity show opioid-specific associations with nonmedical prescription opioid use (NMPOU).

**METHODS:**

Using 2023 Youth Risk Behavior Survey data (N = 20 103), survey-weighted logistic regressions modeled 7 composite predictors. Average marginal effects quantified probability-scale associations. A stacked-outcome test compared associations across 5 substances (NMPOU, alcohol, marijuana, cigarettes, cocaine). Karlson-Holm-Breen decomposition quantified polysubstance mediation.

**RESULTS:**

Predictor-substance associations differed significantly across substance types (*P* = .0075). Bullying was uniquely associated with NMPOU (3.8 percentage points [pp], 95% CI: 1.5 to 6; adjusted odds ratio [AOR] = 1.48, 95% CI: 1.18–1.86) and not with any other substance. Suicidality was associated with NMPOU (6.2 pp, 95% CI: 4.2 to 8.3; AOR = 1.89, 95% CI: 1.58–2.26) and marijuana but not alcohol, indicating partial opioid specificity. Polysubstance use had the largest overall association (6.3 pp, 95% CI: 4.1 to 8.5; AOR = 2.09, 95% CI: 1.63–2.66) but reflected general substance use liability. All 7 predictors were independently significant in the fully adjusted model (n = 9401).

**CONCLUSION:**

Bullying and suicidality are associated with opioid-specific hazard, indicating why certain adolescents turn to opioids, whereas polysubstance use, sexual victimization, household adversity, and community safety adversity reflect general substance use liability. Where bullying or suicidality is identified, targeted opioid prevention is warranted; where NMPOU is present, clinicians should screen for bullying victimization and unmet mental health needs.

## Introduction

Nonmedical prescription opioid use (NMPOU) among adolescents remains a serious clinical and public health concern. Prescription pain relievers account for approximately 96.6% of all opioid misuse among people aged 12 years and older.^1^ Approximately 1 in 4 high school seniors has used prescription opioids medically or nonmedically,^2^ and adolescents who misuse these medications are significantly more likely to engage in suicidal behavior, violence, and other substance use.^3,4^ Many adolescents perceive prescription opioids as safer than illicit drugs, initiating use through nonmedical pathways that heighten addiction and overdose risks.^5–8^

Research published in *Pediatrics* (American Academy of Pediatrics [AAP]) has documented several individual risk factors for adolescent NMPOU, including polysubstance involvement, risky behavior, and suicidality.^3,4,9^ However, these same adversities also predict alcohol, marijuana, and other substance use, raising a question that prior work has not resolved: which adversities create opioid-specific hazard, and which reflect general substance use liability? Answering this question is essential for prevention. Opioid-specific approaches emphasize trauma-informed care for social pain, peer-mediated antibullying programs, and primary care–embedded distress-tolerance support targeting the social-rejection-to-opioid-receptor circuit. Universal substance-use approaches, by contrast, emphasize broad refusal-skills curricula; screening, brief intervention, and referral to treatment (SBIRT); and family monitoring across all substances, with weaker leverage on opioid-specific affective pathways.^10^

Neuroscience offers a candidate mechanism for opioid specificity. Social Pain Overlap Theory (SPOT) provides a neurobiological explanation: social rejection activates the same mu-opioid receptor circuits that process physical pain,^11–13^ and naltrexone blockade of this system—meaning when naltrexone temporarily blocks brain opioid receptors in healthy adults, people report briefly feeling less socially connected to others—reduces feelings of social connection.^14,15^ Social deprivation disrupts endogenous opioid and endocannabinoid signaling,^16,17^ creating neurobiological vulnerability specifically to opioid use. If social pain drives opioid selection, distress-related adversities should associate more strongly with opioids than with other substances.

To test this, the present study organizes different theories spanning developmental psychology, neuroscience, and social ecology into 3 empirically testable frameworks, each generating distinct predictions about opioid specificity. Framework 1 (F1: Generalized Risk-Taking) proposes that NMPOU reflects shared externalizing liability: adolescents predisposed to reward-seeking engage in multiple risk behaviors, with no pharmacologically specific pathway to any single substance.^18–23^ F1 predicts polysubstance involvement as the strongest predictor with no opioid-specific signal; Jones et al^23^ found NMPOU associated with virtually all surveyed risk behaviors, consistent with F1. Framework 2 (F2: Distress Relief) proposes that opioids provide pharmacologically specific relief for emotional suffering, consistent with the Self-Medication Hypothesis and its identification of drug-affect specificity.^24^ Prior research has linked suicidality^25–27^ and bullying^28,29^ to NMPOU, but whether these associations are opioid specific has not been tested. Bullying takes verbal, physical, relational, and electronic forms, and prior longitudinal work shows cybervictimization contributes incrementally to distress outcomes beyond face-to-face victimization^30^; the 2023 Youth Risk Behavior Survey (YRBS) captures school bullying and cyberbullying but not finer-grained subtypes. Framework 3 (F3: Contextual Adversity) locates NMPOU within cumulative environmental exposure, operationalized in this study with 3 measured composites: sexual victimization, household adversity (parental intimate partner violence, household substance use, household mental illness), and community safety adversity (school-safety absences, weapon threats at school, neighborhood violence). The adverse childhood experiences (ACE) framework demonstrated dose-response relationships between adversity and substance use,^31^ with 2023 YRBS data attributing 84.3% of the population attributable fraction for prescription opioid misuse to ACEs.^32^ Sexual victimization^33,34^ and household adversity produce overlapping risk, but their opioid specificity has not been tested. The 2023 YRBS is the first national dataset to include household adversity items,^35^ enabling a direct test. The Social Ecological Model^36^ and Developmental Psychopathology^37,38^ inform the analytic architecture; differential exposure to community adversity shapes adolescent opioid risk environments.^39–41^

Using 2023 YRBS data, this study evaluates these 3 frameworks. A stacked-outcome heterogeneity test compares predictor-substance associations across 5 substance types to determine opioid specificity. Karlson-Holm-Breen (KHB) decomposition quantifies the proportion of each association mediated by polysubstance involvement. F1 predicts that polysubstance involvement should be the strongest NMPOU predictor and that no predictor should show substance-specific associations.^18^ F2 predicts that distress variables (bullying, suicidality) should show stronger associations with NMPOU than with other substances and should remain significant after polysubstance adjustment.^24^ F3 predicts that adding contextual adversity should substantially reduce individual-level distress associations.^31^ Building upon foundational research that documented these risk factors individually,^3,4,9^ the central question is whether any adversity is opioid specific or whether all associations reflect general substance use liability.

## Methods

### Data Source and Design

Data come from the 2023 YRBS, a biennial, nationally representative survey of US high school students (grades 9–12) administered by the Centers for Disease Control and Prevention (CDC).^35,42,43^ Sampling used a 3-stage cluster design (counties, schools, classrooms); 20 103 valid questionnaires were obtained (school response rate 76%). Participation was voluntary and anonymous and required parental consent; sample weights adjusted for nonresponse and oversampling.^44^ The IRB of University of Arizona (STUDY00007753) classified this analysis as not human subjects research.

Grades 9 to 12 (ages approximately 14–18) constitute the developmental window in which NMPOU initiation rises most rapidly and approaches its lifetime peak in early adulthood at ages^19–22,45^ corresponding to limbic reward maturation outpacing prefrontal regulation^19^ and elevated hypothalamic-pituitary-adrenal (HPA)-axis reactivity to social-evaluative stress. Mid-to-late adolescence is also the period of greatest peer-context salience and peak bullying and suicidality prevalence in US surveillance data, motivating focused analysis at this developmental stage rather than at early adolescence.

### Measurement

The primary outcome was lifetime NMPOU (Q49: “how many times have you taken prescription pain medicine without a doctor’s prescription or differently than how a doctor told you to use it?”), coded dichotomously: 1 = any lifetime endorsement (Q49 response options B-F: 1 or more times) and 0 = no endorsement (response A: 0 times); weighted prevalence 11.6%. Past-30-day NMPOU was assessed in sensitivity analysis SM9 using Q92 (verbatim): “During the past 30 days, how many times did you take prescription pain medicine without a doctor’s prescription or differently than how a doctor told you to use it?” (weighted prevalence 4.3%).

Seven composite predictors, each coded dichotomously (0 = no items endorsed; 1 = any item endorsed), spanned 3 frameworks. Details are in Supplemental Tables 4 and 10.^46^ Framework 1 (Generalized Risk-Taking): lifetime polysubstance use (cigarettes, alcohol, marijuana; Q31, Q41, Q46; alpha = 0.88) and risk behavior (weapon carrying, physical fighting; Q12, Q17; Spearman-Brown = 0.63). A 3-item version adding riding with a drunk driver (Q10) was tested in sensitivity analysis SM7; Q10 was excluded because it captures exposure to others’ substance use rather than the adolescent’s own behavior. Framework 2 (Distress Relief): bullying victimization (school and cyber; Q24, Q25; Spearman-Brown = 0.87) and suicidality (ideation, plan, attempt; Q27-Q29; alpha = 0.94). Framework 3 (Contextual Adversity): sexual victimization (forced intercourse, early sexual debut at age 12 or younger; Q19, Q57; Spearman-Brown = 0.75), household adversity (parental intimate partner violence, household substance use, household mental illness; Q91, Q100, Q101; alpha = 0.81), and community safety adversity (missed school days due to safety concerns at school or in transit (Q14, past 30 days); weapon threats on school property (Q15, past 12 months); witnessing neighborhood violence (Q18, lifetime); alpha = 0.66), a formative index (Supplemental Table 9). Early sexual debut at age 12 or younger is widely interpreted as a marker of sexual victimization rather than precocity.^47^ Six independent tests showed its substance associations are general rather than opioid specific (Supplemental Table 8). Household adversity and community safety items were national-module only, producing approximately 30% to 34% structural missingness; sexual victimization items were administered to the full sample. Demographic covariates included sex, grade, and race and ethnicity (white [reference], Other, Black, Latine/Multiple Latine, Multiple Non-Latine). The CDC race and ethnicity variable classifies Hispanic/Latino ethnicity as taking precedence over race.^48^

### Analytic Samples

A 2-sample strategy addressed structural missingness^49^: the full sample (N = 20 103) was used where standard-version items were available, and the national-module subsample (n = 13 974) was used for models requiring household and community adversity composite variables. Subsample comparability was confirmed (all adjusted odds ratio [AOR] changes <4.3%; Supplemental Table 10).

### Statistical Analysis

All analyses incorporated survey weights, strata, and primary sampling units using the survey package in R 4.5.2. Set A (Total Association) models estimated unconditional associations: M1 (demographics), M2a (+ F2 distress: bullying, suicidality), M2b (+ F3 adversity: sexual victimization, household adversity, community safety). Set B (Conditional Specificity) models added polysubstance use: M3 (+ F1 + F2, full sample), M4 (+ sexual victimization and household adversity, subsample), M5 (+ community safety, subsample). Block-wise Wald tests evaluated incremental model improvement.

Average marginal effects (AMEs), computed via the marginaleffects package,^50^ quantified associations on the probability scale.^51^ AORs are reported alongside AMEs for comparability with prior literature (Supplemental Tables 5–7). To test whether each predictor was more strongly associated with opioids than with other substances, a stacked-outcome heterogeneity test restructured data so each student appeared 5 times (1 per substance: NMPOU, alcohol, marijuana, cigarettes, cocaine), with leave-one-out polysubstance composites to avoid circularity.^51^ Predictor-by-outcome-type interactions tested whether associations differ across substances (NMPOU as reference); a joint Wald test evaluated opioid specificity. A confirmatory 3-substance model (NMPOU, alcohol, marijuana) was also estimated (see Supplemental Methods for detailed descriptions of these analytic techniques).^52^

To determine how much of each predictor’s association with opioids operates through general polysubstance involvement, KHB decomposition quantified the proportion mediated, correcting for noncollapsibility of logistic regression coefficients (see Supplemental Methods for a worked example).^53,54^ Results are interpreted as accounting decompositions, not causal mediation.

Eleven sensitivity analyses tested alternative operationalizations (SM1-SM11; Supplemental Information, Supplemental Table 15). A linear probability model (LPM) confirmed the stacked-outcome results. Past-30-day NMPOU (Q92) was examined as an alternative outcome. Complete-case analysis was used; item-level nonresponse was 1.8% to 6.7%. Prespecified sensitivity power analysis (Supplemental Methods, Supplemental Table SP1), with the sensitivity power approach following Hoenig and Heisey 2001,^55^ Demidenko 2007,^56^ and Hsieh, Bloch, and Larsen 1998,^57^confirmed the full ecological model (M5; n = 9401) had greater than or equal to 80% power to detect AORs greater than or equal to 1.29 to 1.51 at alpha = 0.05; the underpowered cocaine comparison (n = 299 events) is addressed in Limitation 7.

Generative artificial intelligence (Claude Opus 4.6, Anthropic) was used to assist with manuscript editing, code documentation, and formatting for journal guidelines.

## Results

Lifetime NMPOU prevalence was 11.6% (N = 20 103). Table 1 presents survey-weighted prevalence by demographics and composite predictor status.

Figure 1 presents AMEs from the full ecological model (M5; n = 9401; Supplemental Information, Supplemental Table 5). An AME represents the average change in predicted NMPOU probability when a predictor switches from absent to present, expressed in percentage points (pp). For instance, a polysubstance use AME of 6.3 pp indicates a 6.3-pp-higher NMPOU probability for an adolescent who has used other substances, holding other predictors constant. Unlike odds ratios, AMEs are directly interpretable as probability differences and unaffected by noncollapsibility.^58^ All 7 composite predictors were independently significant. Effect sizes ranged from 3.0 pp (household adversity) to 6.3 pp (polysubstance use); all AOR and 95% CI values appear in Figure 1 and Supplemental Table 5. In Set A, all F2 variables were strongly associated with NMPOU (M2a; Supplemental Table 5). Adding F1 in Set B (M3) attenuated but did not eliminate distress associations. Block-wise Wald tests confirmed significant incremental improvement at each step (all *P* < .0001). Cumulative attenuation from M3 subsample to M5 was 36.5% for bullying and 21.1% for suicidality (Supplemental Table 13), indicating partial operation through shared contextual adversity, although most of each association remained.

Figure 2 compares AMEs for all 7 composite predictors across 5 substance outcomes (Supplemental Tables 6 and 7). Polysubstance use had the largest association across all substances (6.3 pp, 95% CI: 4.1 to 8.5), consistent with F1 (F1 supported: generalized risk-taking). Risk behavior (4.1 pp, 95% CI: 1.4 to 6.8) was associated with NMPOU but showed no significant heterogeneity across substances (*P* =.0327). Bullying increased NMPOU probability by 3.8 pp (95% CI: 1.5 to 6) but was not significant for any other substance, consistent with opioid specificity (F2 supported: bullying opioid specific). Suicidality was associated with NMPOU (6.2 pp, 95% CI: 4.2 to 8.3) and marijuana but not alcohol, showing partial specificity (F2 partially supported: suicidality). Sexual victimization showed larger associations for cocaine and marijuana than for NMPOU (3.1 pp, 95% CI: 0.0 to 6.3), consistent with general rather than opioid-specific adversity (F3 partially supported: general not specific). Household adversity (3.0 pp, 95% CI: 1.1 to 4.9) and community safety adversity (5.2 pp, 95% CI: 3.0 to 7.4) were significant for NMPOU and several comparison substances. The stacked-outcome heterogeneity test confirmed significant differences across substance types (F = 4.61, *P* =.0075; Supplemental Table 12). A linear probability model yielded consistent results (F2 distress interaction F = 11.28, *P* =.0001). Suicidality (*P* =.0101) and sexual victimization (*P* =.0194) showed significant heterogeneity; bullying showed marginal heterogeneity in the 5-substance model (*P* =.1612) but clear significance in the 3-substance model (F = 8.24, *P* =.0002).

KHB decomposition (Supplemental Table 11) showed that only 10.3% of bullying’s and 15.3% of suicidality’s total NMPOU associations were mediated by polysubstance involvement. Sexual victimization had the highest mediation of all 6 predictors (25.2%; 95% CI: 11.4%–39.0%), meaning more than one-quarter of its total NMPOU association was channeled through general polysubstance involvement, consistent with an adversity-exposure pathway rather than a direct opioid-specific mechanism. Sensitivity analyses (Supplemental Table 15) reinforced primary findings: past-30-day NMPOU (Q92) replicated the pattern; persistent sadness (S1) yielded a comparable AOR; suicidality’s association was minimally attenuated by adding forced sexual intercourse (S3: 4.4%). Household adversity, polysubstance use, and risk behavior showed comparable direction and magnitude to primary M5 estimates across sensitivity models.

Framework endorsement was defined as endorsing at least 1 item within any composite measure of that framework’s predictor set. Figure 3 displays framework endorsement among NMPOU-positive adolescents by race and ethnicity. In Panel A, F1 endorsement alone was high across all groups: 64.8% (Latine), 62.5% (white), 57.2% (Black), 54% (Other), and 50.4% (Multiple Non-Latine). In Panel B, F1-F2 co-endorsement was substantial: 46.5% (Latine), 44.4% (white), 30% (Black), 40.3% (Other), and 37.8% (Multiple Non-Latine). Panel C adds F3 (contextual adversity). The 3-way F1-F2-F3 intersection was the largest overlapping region in most groups (42.3% Latine, 38.9% white, 26.6% Black, 37% Multiple Non-Latine). From Panel B to Panel C, nearly all F1-F2 co-endorsement was absorbed into the 3-way intersection, (0.8%–5.5% exclusive F1-F2 remaining). The frameworks identified overlapping risk profiles: the typical NMPOU-positive adolescent endorsed all 3 domains simultaneously. Sex-stratified diagrams appear in Supplemental Figure 1.^38,59^

Composite reliability estimates are reported in Supplemental Information (Supplemental Table 9); subsample validation in Supplemental Table 10.

## Discussion

Foundational research in AAP has documented that adolescents who misuse prescription opioids engage in virtually all surveyed risk behaviors.^3,4,9^ The present study examined whether these associations reflect opioid-specific pathways vs general substance use liability, formally testing predictor-substance specificity across 5 substances.

Lifetime polysubstance use had the largest association with NMPOU probability (6.3 pp), consistent with generalized risk taking (F1) carrying the greatest epidemiologic magnitude. However, distress associations (F2) differed significantly across substance types (*P* =.0075), while F1 and F3 (contextual adversity: sexual victimization, household, and community safety exposures) variables showed broadly similar patterns across substances (Figure 2). Clinically, polysubstance involvement points to where resources should be deployed broadly, whereas opioid-specific distress associations suggest why certain adolescents turn to opioids rather than other substances.

Bullying produced the clearest opioid-specificity signal (3.8 pp), with no association for any other substance. Only 10.3% of the association was mediated by polysubstance involvement, consistent with SPOT.^11,30^ Social deprivation disrupts endogenous opioid signaling, creating reciprocal vulnerability where social exclusion specifically increases opioid seeking.^17^ Suicidality was associated with both NMPOU and marijuana but not with alcohol, indicating partial opioid specificity.^60,61^ Sexual victimization, classified within F3, behaves as a hybrid construct: although it produces emotional distress, 6 independent tests confirmed its substance associations are general rather than opioid specific (Supplemental Table 8). Its 25.2% polysubstance mediation, the highest among predictors, may suggest a general trauma-coping mechanism rather than the social-pain-to-opioid circuit characterizing bullying.^62–66^

Risk behavior (4.1 pp) supported F1, although the finding depended on composite specification (Supplemental Table 15, SM7). Inclusion of contextual adversity (F3) attenuated bullying’s association (36.5%), yet the majority remained, and both household adversity (3 pp) and community safety adversity (5.2 pp) were independently significant, suggesting amplifying rather than purely explanatory roles. Suicidality showed smaller attenuation (21.1%), indicating greater robustness to contextual adjustment. Racial and ethnic subgroup patterns provide structural evidence for this contextual role: among Latine adolescents, who showed elevated NMPOU odds across all 7 models (Supplemental Table 5),^67–69^ nearly all F1-F2 co-endorsement also included F3 (only 4.2% exclusive F1-F2 overlap in Panel C of Figure 3), consistent with disproportionate exposure to household adversity driven by structural stressors including acculturative stress, discrimination, and economic disadvantage.^70^ Racial disparities in opioid prescribing^71,72^ shape additional pathways: white adolescents show lower F3 endorsement alongside high F1-F2 overlap, consistent with prescription-access pathways independent of contextual adversity, whereas Black adolescents show lower NMPOU prevalence,^69^ consistent with reduced diversion pathways; among those who do endorse NMPOU, concentration at the F1-F2 intersection aligns with evidence linking NMPOU to suicidality,^73^ suggesting distress pathways play a larger role when prescription access is constrained. Multiple Non-Latine adolescents showed the highest NMPOU prevalence in Table 1, consistent with elevated substance use risk through identity-related stress—defined as the cumulative psychosocial strain that multiracial youth may experience from racial identity ambiguity, exposure to discrimination from multiple racial reference groups, and pressure to navigate competing cultural expectations.^74–76^ Future research should examine culturally specific pathways.^68,69^

As Figure 3 shows, the frameworks describe overlapping risk profiles. Antibullying programs such as Sources of Strength^77^ directly address the unique opioid pathway. Sources of Strength reduces social-rejection exposure at its source by training peer leaders to strengthen relational connectedness and protective network ties, thereby blunting the upstream social-pain input to the mu-opioid receptor circuit hypothesized by SPOT.^77^ Bullying rarely occurs in isolation from polysubstance use and contextual adversity. Prevention must therefore integrate antibullying programs with standardized substance use screening (CRAFFT,^78^ SBIRT^10^) and suicide risk assessment (ASQ^79^) at every adolescent primary-care or well-visit encounter, alongside community-level interventions such as Communities That Care^80^ (a school-community coalition model that selects empirically supported preventive programs to target locally documented risk factors) and PROSPER^81^ (a 6-year community-university partnership delivering family and classroom-based curricula) that address household and community adversity. When bullying or suicidality is present, the social-pain-to-opioid pathway implies heightened analgesic drive, potentially warranting referral to mental health services emphasizing distress-tolerance and emotion-regulation skills (eg, DBT-informed approaches) and family-based counseling that addresses both adolescent emotion regulation and safe household opioid storage; in postsurgical settings, screening for these risk factors may identify adolescents at elevated risk for nonmedical continuation of prescription opioid use.^82^

Strengths include nationally representative data, prespecified analytic rules, formal stacked-outcome tests of substance specificity, composite reliability assessment, and KHB-corrected mediation decomposition.

### Construct Validity

Two limitations bound the construct precision of our SPOT-related inferences. (1) The absence of measures for ongoing physical pain and prior medical opioid exposure^83,84^ means the bullying-NMPOU association cannot be cleanly attributed to the social-pain mechanism rather than to analgesic motivation for ongoing nociceptive pain; future studies pairing validated pediatric pain inventories with electronic health record–linked opioid prescription histories would directly distinguish these pathways. (2) The risk-behavior composite was sensitive to item specification (Supplemental Table 15, SM7); future composite-validation work using item-response-theory methods could yield a more robust risk-behavior measure.

### Internal Validity and Causality

Two limitations bound causal directionality. (3) The cross-sectional design means directional interpretation of the bullying-NMPOU pathway should remain provisional, because NMPOU could also precede bullying through stigma and social withdrawal; future longitudinal cohorts with repeated bullying, suicidality, and NMPOU measurements would clarify temporal ordering. (4) Residual confounding from unmeasured factors—including access to mental health care—could partially explain the observed associations; future quasi-experimental or instrumental-variable designs would tighten causal inference under unmeasured-confounding sensitivity bounds.

### External Validity and Generalizability

Three limitations constrain generalizability. (5) Structural missingness (~30%–34%) in the national-module subsample restricts the F3 inference base; future analyses applying multiple-imputation methods robust to missing-not-at-random patterns would expand the inferential population. (6) The aggregation of Asian, American Indian/Alaska Native, and Native Hawaiian/Pacific Islander adolescents into the “Other” race and ethnicity category can mask heterogeneous risk profiles^76,85–88^; future surveillance with oversampled subgroups (per Saraiya 2024)^88^ would resolve this aggregation. (7) The underpowered cocaine comparison (n = 299 events) constrained the precision of opioid-vs-cocaine specificity contrasts (Supplemental Methods, Supplemental Table P2); future analyses pooling multiple YRBS cycles would increase rare-substance event counts.

Across the 3 validity considerations, primary inferences relied on AMEs on the probability scale (Figures 1 and 2) with consistent LPM corroboration; logistic-coefficient comparisons across substances may nonetheless be affected by residual variance differences.^51^

## Conclusion

Among US high school students, all 7 predictors spanning 3 theoretical frameworks were independently associated with lifetime NMPOU, but they functioned differently. Polysubstance use had the largest overall association (6.3 percentage points), yet bullying was uniquely associated with NMPOU (3.8 pp) and not with any other substance, providing evidence consistent with an opioid-specific distress pathway: the social-pain-to-opioid-relief mechanism. These findings support bidirectional clinical screening: where bullying or suicidality is identified, targeted opioid prevention and counseling are warranted; where NMPOU is present, clinicians could screen for bullying victimization and unmet mental health needs. Because the 3 frameworks describe overlapping rather than distinct risk profiles, prevention should integrate antibullying and suicide prevention programs for opioid-specific pathways with substance use screening and community safety interventions for generalized and contextual risk. Formally testing whether these theoretical frameworks function as competing vs complementary explanatory models across diverse populations represents a logical next analytic step. Future research should also examine whether these risk profiles differ across stages of opioid involvement, from experimentation to repeated misuse.

## Supplementary Material

Supplementary Material

Supplementary Figure 1

## Figures and Tables

**FIGURE 1.**
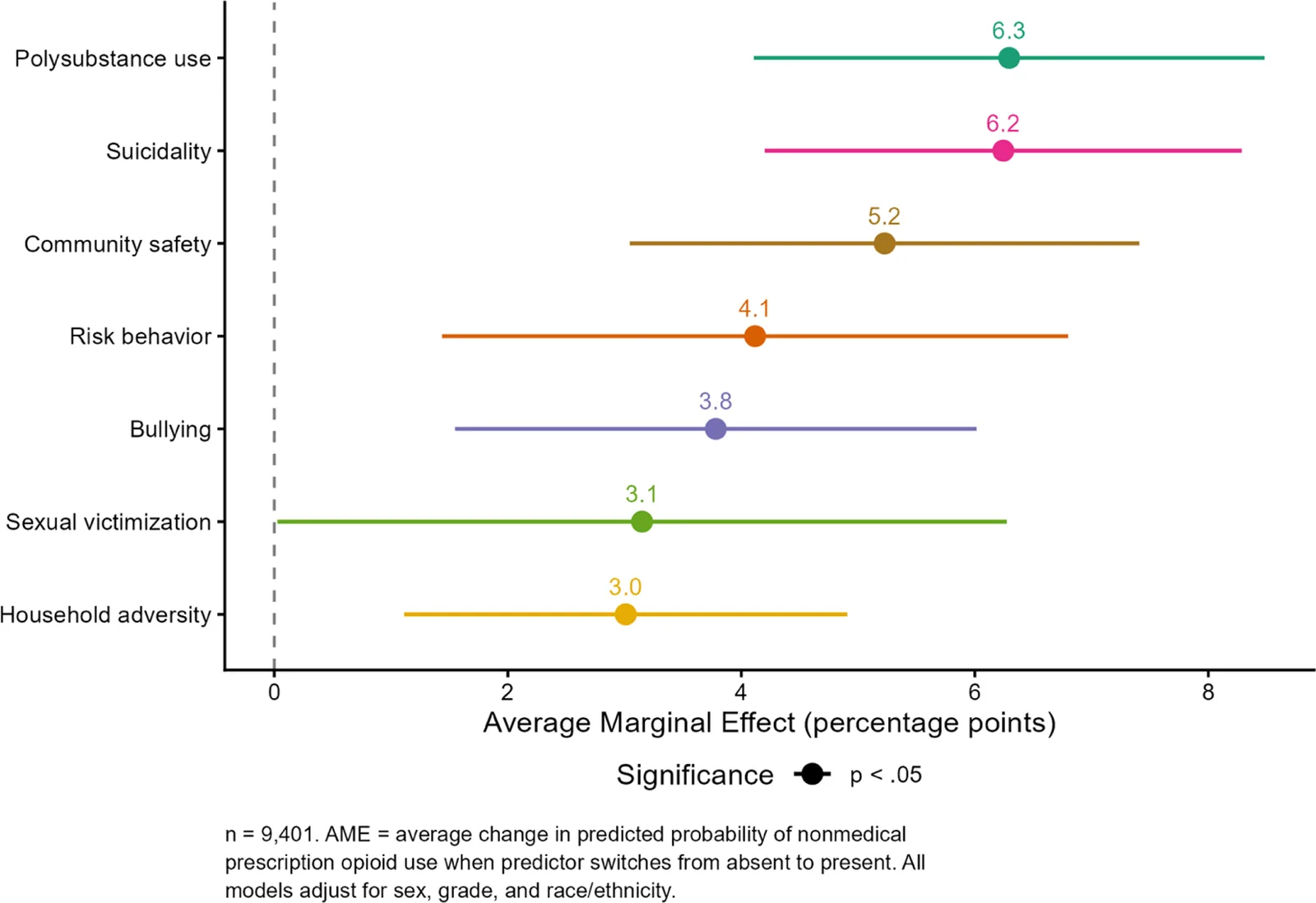
Average marginal effects (percentage points) from the full ecological model (M5) for 7 composite predictors of lifetime nonmedical prescription opioid use. Filled circles indicate *P* < .05.

**FIGURE 2.**
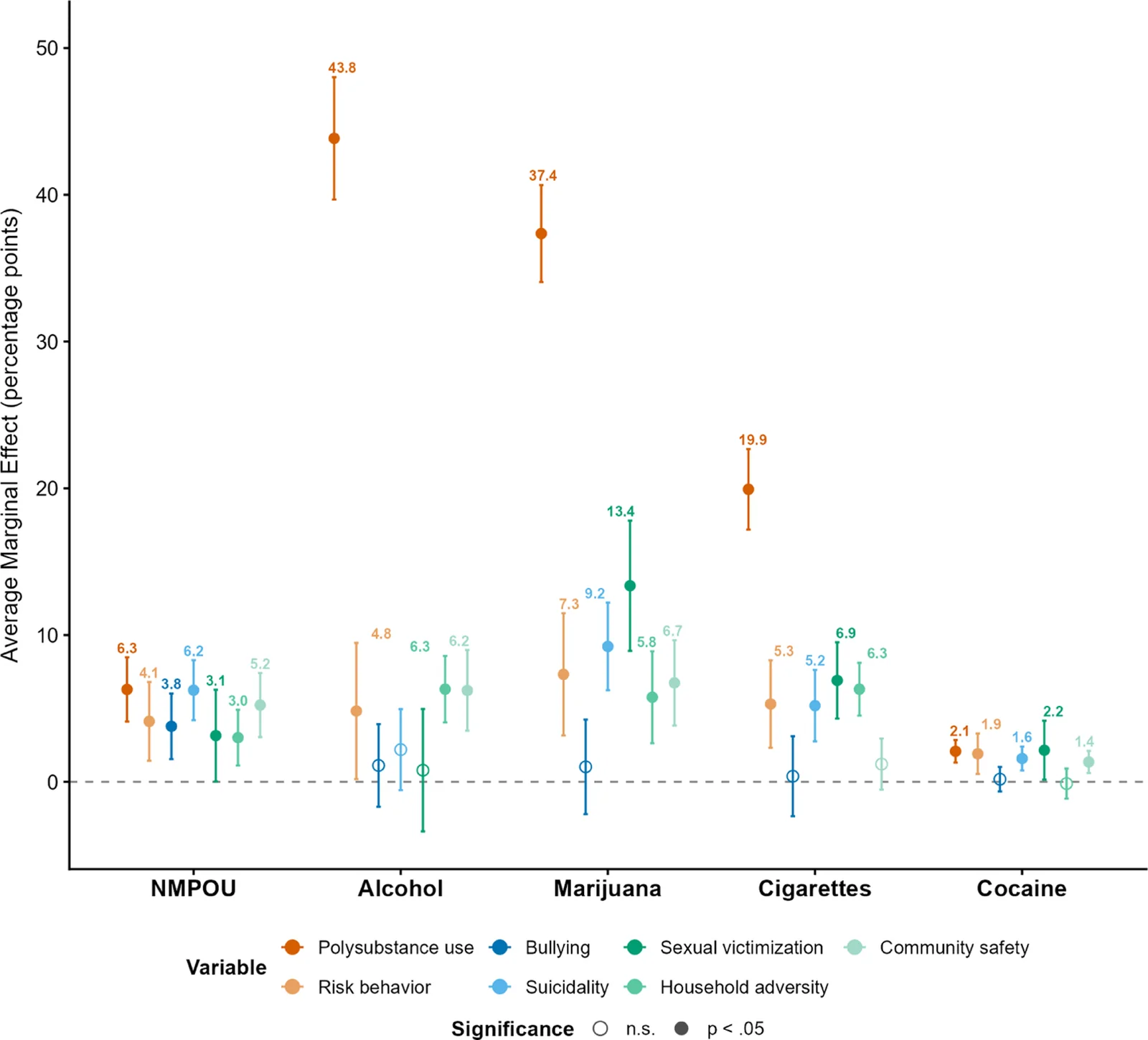
Average marginal effects (percentage points) for 7 composite predictors across 5 substance outcomes (nonmedical prescription opioid use, alcohol, marijuana, cigarettes, cocaine). Each substance model uses leave-one-out polysubstance composites. Filled circles indicate *P* < .05; open circles indicate nonsignificant associations. Abbreviations: AME, average marginal effect; NMPOU, nonmedical prescription opioid use.

**FIGURE 3.**
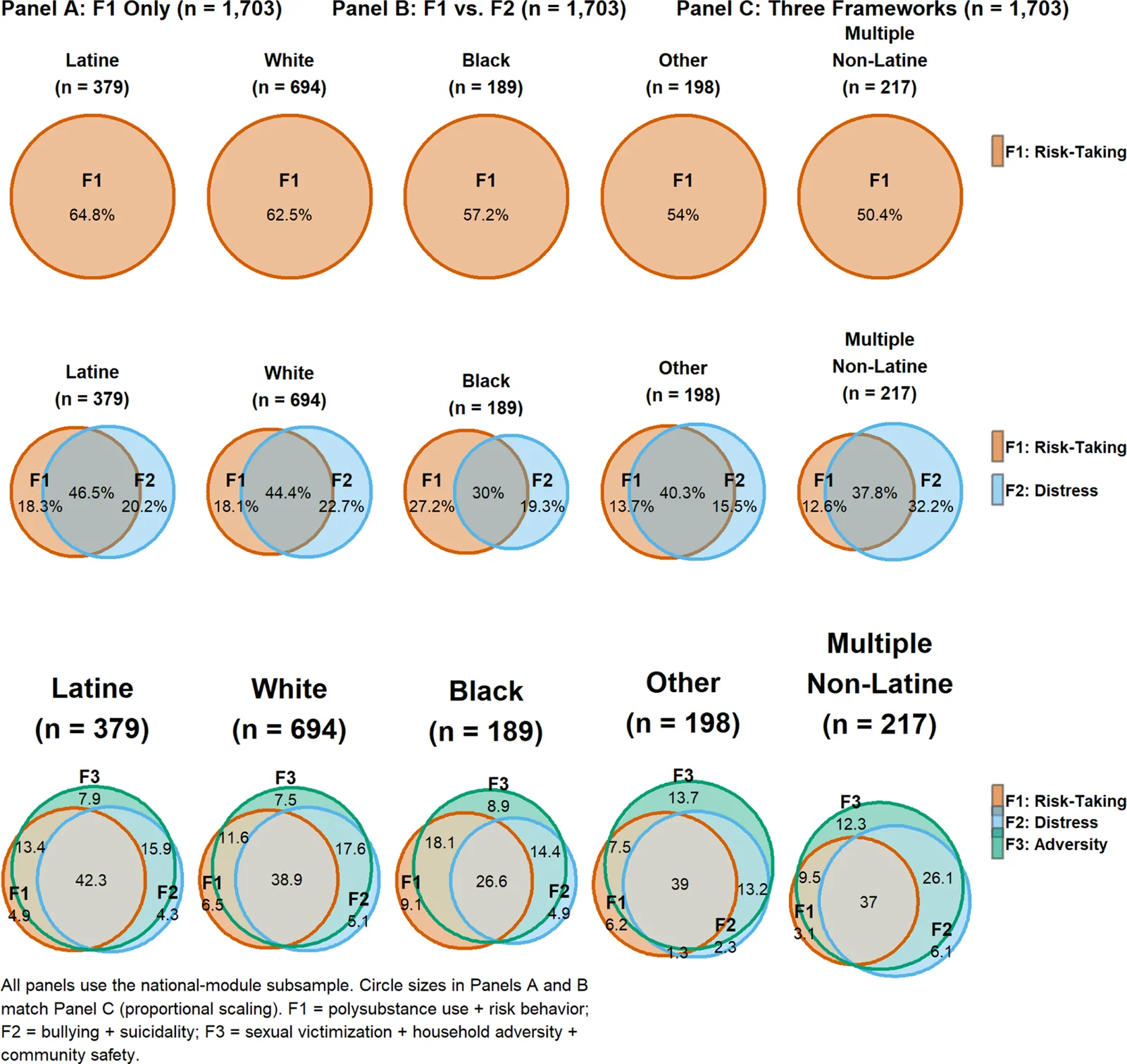
Survey-weighted framework endorsement among nonmedical prescription opioid use–positive adolescents by race and ethnicity. Panel A: Framework 1 (generalized risk-taking) only. Panel B: Framework 1 vs Framework 2 (bullying, suicidality). Panel C: Three-framework Euler diagrams (Framework 1, Framework 2, Framework 3; Framework 3 includes sexual victimization, household adversity, and community safety).

**TABLE 1.** Survey-Weighted Prevalence of Lifetime NMPOU by Demographic Characteristics and Composite Predictor Status

Characteristic	n	Weighted %	Missing (%)	NMPOU Prevalence (%)	95% CI
Overall	19 527		2.9	11.6	10.3–12.9
Sex			0.8		
Female	9884	48.1		13.8	12.1–15.6
Male	10 061	51.9		9.3	8.3–10.4
Race and ethnicity			1.8		
White	9700	48.1		9.9	8.3–11.4
Black	1791	13.3		12.2	10.4–14.1
Latine/multiple Latine	3994	27.4		13.2	11.0–15.4
Multiple non-Latine	1814	6.1		14.4	10.5–18.3
Other	2434	5.0		10.3	6.5–14.1
Grade			1.2		
9th	5680	26.5		12.1	10.5–13.7
10th	5410	25.8		13.0	11.3–14.7
11th	4811	24.3		10.4	8.6–12.2
12th	3961	23.4		10.2	8.0–12.3
Lifetime polysubstance use			21.5		
No	7325	48.4		5.9	5.1–6.7
Yes	8465	51.6		15.3	13.5–17.1
Risk behavior			13.7		
No	15 426	89.5		9.9	8.9–10.8
Yes	1915	10.5		22.3	18.5–26.1
Bullying victimization			1.6		
No	14 619	75.3		7.9	7.0–8.8
Yes	5156	24.7		20.6	18.4–22.9
Suicidality			12.4		
No	12 999	75.7		7.0	6.4–7.7
Yes	4604	24.3		23.4	21.0–25.8
Sexual victimization			18.7		
No	14 584	90.4		9.2	8.3–10.1
Yes	1769	9.6		25.1	21.7–28.5
Household adversity			6.8		
No	6749	56.5		6.5	5.5–7.5
Yes	6275	43.5		16.9	15.0–18.8
Community safety adversity			18.6		
No	7246	65.9		6.7	5.8–7.6
Yes	4134	34.1		19.1	17.2–20.9

Abbreviation: NMPOU, nonmedical prescription opioid use.

Unweighted Ns and survey-weighted percentages are reported per Centers for Disease Control and Prevention guidance. Youth Risk Behavior Survey weights are calibrated for proportion estimation rather than population enumeration. 95% CI = Wald-type confidence interval.

a Household adversity and community safety adversity were administered only on the national questionnaire module (subsample n = 13 974); all other variables were computed on the full sample (N = 20 103). Lifetime polysubstance use = any cigarette, alcohol, or marijuana use (Q31, Q41, Q46). Risk behavior = weapon carrying or physical fighting (Q12, Q17). Bullying victimization = school or electronic bullying (Q24, Q25). Suicidality = seriously considered, planned, or attempted suicide (Q27, Q28, Q29). Sexual victimization = forced sexual intercourse or sexual debut at age 12 or younger (Q19, Q57). Household adversity = parental intimate partner violence, household substance use, or household mental illness (Q91, Q100, Q101). Community safety adversity = unsafe school absences, weapon threats at school, or unsafe neighborhood (Q14, Q15, Q18).

## WHAT’S KNOWN ON THIS SUBJECT:

Adolescent nonmedical prescription opioid use (NMPOU) has been linked to bullying, suicidality, sexual victimization, and polysubstance use, but it remains unknown whether these risk factors are specific to opioids or simply markers of general substance or contextual risks.

## WHAT THIS STUDY ADDS:

Bullying uniquely predicted nonmedical prescription opioid use (not alcohol, marijuana, cigarettes, or cocaine), suggesting opioid-specific hazard consistent with social-pain neurobiology. Suicidality showed partial opioid specificity. Prevention should integrate antibullying and suicide screening with substance use and community safety interventions.

## Abbreviations

AAP: American Academy of Pediatrics
ACE: adverse childhood experience
AME: average marginal effect
AOR: adjusted odds ratio
CDC: Centers for Disease Control and Prevention
F1: Framework 1 (Generalized Risk-Taking)
F2: Framework 2 (Distress Relief)
F3: Framework 3 (Contextual Adversity)
KHB: Karlson-Holm-Breen
LPM: linear probability model
NMPOU: nonmedical prescription opioid use
SBIRT: screening, brief intervention, and referral to treatment
SPOT: Social Pain Overlap Theory
YRBS: Youth Risk Behavior Survey
