# Supplementary material for: Self-Medicating Social Pain? Bullying, Suicidality, and Opioid-Specific Risk in US Adolescents
Source: Pediatr Open Sci. Author manuscript; Available in PMC 2026 Nov 4. (PMC13730214; doi:10.1542/pedsos.2026-001614)
Supplement: Supplementary file 1 [file NIHMS2208976-supplement-Supplementary_Material.docx]

Supplementary Material

### Supplementary Methods

Seven composite predictors were constructed from YRBS items, each coded dichotomously (0 = no items endorsed; 1 = any item endorsed). Composite construction followed the “any endorsed” approach because the goal was to flag exposure presence rather than severity; this approach is standard in YRBS analyses and minimizes missingness.

**Framework 1 (Generalized Risk-Taking).** *Lifetime polysubstance use* combined three items: lifetime cigarette use (Q31: “have you ever tried cigarette smoking, even one or two puffs?”), lifetime alcohol use (Q41: “during your life, on how many days have you had at least one drink of alcohol?”), and lifetime marijuana use (Q46: “during your life, how many times have you used marijuana?”). All items were dichotomized (any use vs. none). Tetrachoric alpha = 0.88. *Risk behavior* (primary, 2-item) combined weapon carrying (Q12: “during the past 30 days, on how many days did you carry a weapon such as a gun, knife, or club?”) and physical fighting (Q17: “during the past 12 months, how many times were you in a physical fight?”). Spearman-Brown = 0.63. A three-item version adding riding with a drunk driver (Q10) was tested in sensitivity analysis SM7.

**Framework 2 (Distress Relief).** *Bullying victimization* combined school bullying (Q24: past 12 months) and cyberbullying (Q25: past 12 months). Spearman-Brown = 0.87. *Suicidality* combined ideation (Q27), plan (Q28), and attempt (Q29), all past 12 months. Tetrachoric alpha = 0.94.

**Framework 3 (Contextual Adversity).** *Sexual victimization* combined forced sexual intercourse (Q19: lifetime) and early sexual debut at age 12 or younger (Q57, dichotomized). Early debut at this threshold is widely interpreted as a marker of sexual victimization rather than precocity. Spearman-Brown = 0.75. Although sexual victimization produces emotional distress, it was classified within F3 (Contextual Adversity) rather than F2 (Distress Relief) based on two considerations. First, the YRBS forced-intercourse (Q19) and early-debut (Q57) items do not specify perpetrator identity, precluding attribution to peer-based social pain (the interpersonal mechanism theorized by SPOT, which requires social rejection by known peers) versus other trauma sources such as familial or stranger-perpetrated violence. Second, six independent empirical tests (Table S8) confirmed that sexual victimization’s substance associations are general rather than opioid-specific: it was significantly associated with 4 of 5 substances, showed the highest polysubstance mediation of all predictors (25.2%), and had an opioid specificity ratio below 1.0. These patterns are consistent with broad negative-reinforcement trauma-coping (in which any aversive state triggers drug-seeking) rather than the social-pain-to-opioid circuit that characterizes bullying. *Household adversity* combined parental intimate partner violence (Q91), household substance use (Q100), and household mental illness (Q101), all past 12 months, national-module only. Tetrachoric alpha = 0.81. *Community safety adversity* combined unsafe school absences (Q14), weapon threats at school (Q15: past 12 months), and unsafe neighborhood (Q18: past 12 months), national-module only. Tetrachoric alpha = 0.66; however, as a formative index, high intercorrelation is not expected. Pairwise tetrachoric correlations were: Q14-Q15 r = 0.47, Q14-Q18 r = 0.32, Q15-Q18 r = 0.39; the heterogeneous magnitudes are consistent with a formative (causal indicator) composite rather than a reflective scale. Framework 3 items were administered only to the national-module subsample (n = 13,974); reliability was therefore computed on national-module respondents only. Survey weights remain valid for this subsample because the YRBS weighting scheme was designed to produce valid estimates for both the full sample and the national-module subsample.

Internal consistency was evaluated using tetrachoric-based metrics appropriate for dichotomous items. Tetrachoric alpha and McDonald’s omega (WLSMV estimation) were computed for composites with three or more items, and the Spearman-Brown prophecy formula applied to the tetrachoric correlation for two-item composites. Percentile bootstrap confidence intervals (500 resamples) were computed for the primary metric of each composite and are reported in Table S9. Because composites were dichotomous “any exposure” indicators, internal consistency is reported for transparency and does not imply unidimensional latent scaling. The risk behavior composite (Spearman-Brown = 0.63, tetrachoric r = 0.46) reflects the modest tetrachoric correlation between weapon carrying and physical fighting; this is interpreted as a pragmatic behavioral marker rather than a reliable scale. Community safety adversity (alpha = 0.66) is operationalized as a formative index (any safety adversity) rather than a reflective scale; the items represent distinct environmental hazards, and high intercorrelation is not expected. Full reliability statistics are reported in Table S9.

### Analytic Techniques Explained

**Stacked-Outcome Heterogeneity Test.** To test whether predictor-substance associations differ across substance types, the dataset was restructured so each student contributed five rows (one per substance outcome: NMPOU, alcohol, marijuana, cigarettes, cocaine). Survey weights were divided by five (or three, in the confirmatory model) to maintain correct population totals, and the survey design specified two-level clustering (PSU and student) to account for the non-independence of rows from the same student. Each row contained the same predictor values but a different outcome indicator. To avoid circularity in the polysubstance composite, leave-one-out (LOO) composites excluded whichever substance served as that row’s outcome and substituted NMPOU. The stacked model included main effects for all predictors plus predictor-by-outcome-type interaction terms (NMPOU as reference). A negative interaction coefficient means the association is stronger for NMPOU than for the comparison substance. A joint Wald test on the interaction terms provides a single omnibus p-value for heterogeneity.

**KHB Decomposition.** The Karlson-Holm-Breen method decomposes a predictor’s total association into direct and indirect components, correcting for the noncollapsibility of logistic regression odds ratios. The three-stage procedure: (1) regress the mediator (lifetime polysubstance use) on the predictor plus all other covariates via survey-weighted linear regression (gaussian family), saving the residuals; (2a) fit the full survey-weighted logistic model with the mediator included (coefficient = direct effect); (2b) fit a second logistic model replacing the mediator with its residuals (coefficient = total effect). The indirect effect equals total minus direct on the log-odds scale; the percentage mediated equals indirect divided by total times 100. For example, if bullying’s total log-odds coefficient is 0.50 and its direct coefficient is 0.45, the indirect component is 0.05, and 10% of the total association is accounted for by polysubstance involvement.

**Interaction Testing.** Separate Wald tests evaluated each predictor’s interaction with outcome type (e.g., bullying-by-outcome-type), testing whether that specific predictor’s associations differ across substances. A joint Wald test combining F2 distress interactions (bullying, suicidality) provided the omnibus test of distress-specific heterogeneity; sexual victimization was tested separately as an F3 variable.

**Confirmatory Three-Substance Model.** Because cigarettes and cocaine may introduce noise (low prevalence, different pharmacological profiles), a three-substance model (NMPOU, alcohol, marijuana) provided a confirmatory test of opioid specificity using the most commonly used adolescent substances.

### Sensitivity Power Analysis

We computed sensitivity power rather than observed or post-hoc power, asking: given the achieved analytic sample sizes and the YRBS three-stage cluster design, what was the smallest effect size detectable at alpha = 0.05 two-sided with 80% power? Observed power computed from the realized p-value is a deterministic function of the test statistic and is uninformative;^55^ sensitivity power addresses the inferentially relevant question of design adequacy.

For each focal predictor in the full ecological model (M5; n = 9,401), the analytical minimum detectable effect size (MDES) on the adjusted odds ratio (AOR) scale was computed as MDES = exp(SE x (Z_alpha + Z_beta)), where SE is the design-adjusted standard error of the log-AOR coefficient from survey-weighted logistic regression (incorporating clustering, stratification, and survey weights), Z_alpha = 1.96 (two-sided alpha = 0.05), and Z_beta = 0.84 (80% power).^56,57^ The design-adjusted SE captures the full YRBS sampling complexity by construction, so no separate design-effect correction is needed.

For the stacked-outcome five-substance model, MDES was computed identically for each predictor-by-substance interaction term, with NMPOU as the reference level. The interaction MDES is interpreted as the minimum interaction OR -- the departure from the NMPOU-anchored predictor association -- that the stacked test could detect at 80% power.

To validate the analytical MDES, we ran a parametric bootstrap simulation for the headline bullying-NMPOU test in M5. With 500 simulation replicates (random seed = 47) and the bullying log-OR coefficient fixed at log(MDES) = log(1.379), Y was resimulated from the implied Bernoulli model under the actual M5 svydesign, and the M5 svyglm was refit. Empirical power was 0.79 (Monte Carlo standard error approximately 0.018), within Monte Carlo error of the analytical target of 0.80, confirming the Wald-test sensitivity power calculation. R version 4.5.2; survey 4.4.8; marginaleffects 0.32.0.

**M5 sensitivity power (Table SP1).**

At alpha = 0.05 and 80% power, the M5 model could detect adjusted odds ratios as small as 1.29 (suicidality), 1.38 (bullying, household adversity, community safety adversity), 1.41 (polysubstance use, risk behavior), and 1.51 (sexual victimization). All seven predictors had MDES <= 1.51, at or near the moderate clinical-effect threshold of AOR = 1.50 commonly used in pediatric NMPOU literature (McCabe et al. 2017,^2^ Cho et al. 2021,^3^ Bhatia et al. 2020^4^). Observed sensitivity power at the realized effect sizes ranged from 0.58 (sexual victimization) to 1.00 (suicidality, polysubstance use, community safety adversity), indicating adequate power for all primary tests.

**Table SP1. Sensitivity power analysis for M5 predictors.**

| **Predictor** | **Term** | **Observed_AOR** | **Observed_SE_logOR** | **MDES_AOR_80pct** | **Observed_power_at_obs_AOR** |
| --- | --- | --- | --- | --- | --- |
| Bullying | bu_1Yes | 1.483 | 0.1147 | 1.379 | 0.93 |
| Suicidality | sc_1Yes | 1.889 | 0.0911 | 1.291 | 1 |
| Polysubstance use | su_lifeYes | 2.085 | 0.1246 | 1.418 | 1 |
| Risk behavior | rb_1Yes | 1.505 | 0.1232 | 1.412 | 0.913 |
| Sexual victimization | sv_1Yes | 1.377 | 0.1478 | 1.513 | 0.582 |
| Household adversity | hh_1Yes | 1.398 | 0.1147 | 1.379 | 0.832 |
| Community safety adversity | ms_1Yes | 1.744 | 0.1151 | 1.38 | 0.998 |

**Stacked-outcome interaction sensitivity power (Table SP2).**

The MDES for predictor-by-substance interaction ORs varied substantially by substance prevalence. For substances with high prevalence in the analytic sample (alcohol n = 6,319 events; marijuana n = 3,901 events), the bullying-by-substance interaction MDES was 1.55-1.61 -- adequate to detect moderate departures from the NMPOU-anchored bullying association. For cocaine (n = 299 events), the bullying-by-cocaine MDES was 2.27, meaning only interaction ORs >= 2.27 (a 127% departure from the NMPOU bullying association) could be reliably detected. This confirms quantitatively the underpowered cocaine-comparison concern noted in Limitation 7 of the Discussion. The observed bullying-by-cocaine interaction OR was 0.636 (p = 0.146) -- nonsignificant, consistent with both opioid specificity for bullying and with cocaine-test underpower. Future analyses pooling multiple YRBS cycles would increase cocaine event counts and tighten this contrast.

**Table SP2. Sensitivity power analysis for predictor-by-substance interactions.**

| **Term** | **Observed_int_OR** | **SE_log_intOR** | **MDES_int_OR_80** | **p_value** |
| --- | --- | --- | --- | --- |
| outcome_typeAlcohol:bu_1Yes | 0.686 | 0.1568 | 1.552 | 0.0318 |
| outcome_typeMarijuana:bu_1Yes | 0.647 | 0.1706 | 1.613 | 0.0242 |
| outcome_typeCigarettes:bu_1Yes | 0.716 | 0.1979 | 1.741 | 0.115 |
| outcome_typeCocaine:bu_1Yes | 0.636 | 0.2931 | 2.273 | 0.146 |
| outcome_typeAlcohol:sc_1Yes | 0.648 | 0.1284 | 1.433 | 0.005 |
| outcome_typeMarijuana:sc_1Yes | 1.004 | 0.1412 | 1.485 | 0.9777 |
| outcome_typeCigarettes:sc_1Yes | 0.892 | 0.1433 | 1.494 | 0.4398 |
| outcome_typeCocaine:sc_1Yes | 1.286 | 0.2854 | 2.224 | 0.3946 |
| outcome_typeAlcohol:sv_1Yes | 0.887 | 0.2173 | 1.838 | 0.5909 |
| outcome_typeMarijuana:sv_1Yes | 1.923 | 0.202 | 1.761 | 0.0065 |
| outcome_typeCigarettes:sv_1Yes | 1.335 | 0.1667 | 1.595 | 0.1067 |
| outcome_typeCocaine:sv_1Yes | 2.041 | 0.3479 | 2.65 | 0.0611 |
| outcome_typeAlcohol:rb_1Yes | 0.814 | 0.2142 | 1.822 | 0.3544 |
| outcome_typeMarijuana:rb_1Yes | 1.04 | 0.1921 | 1.713 | 0.8426 |
| outcome_typeCigarettes:rb_1Yes | 1.193 | 0.1644 | 1.585 | 0.3035 |
| outcome_typeCocaine:rb_1Yes | 2.011 | 0.297 | 2.298 | 0.0351 |
| outcome_typeAlcohol:hh_1Yes | 1.16 | 0.174 | 1.628 | 0.4098 |
| outcome_typeMarijuana:hh_1Yes | 1.179 | 0.1979 | 1.741 | 0.4214 |
| outcome_typeCigarettes:hh_1Yes | 1.41 | 0.1822 | 1.666 | 0.0821 |
| outcome_typeCocaine:hh_1Yes | 0.699 | 0.3199 | 2.45 | 0.284 |
| outcome_typeAlcohol:ms_1Yes | 0.759 | 0.1547 | 1.543 | 0.0977 |
| outcome_typeMarijuana:ms_1Yes | 0.904 | 0.17 | 1.61 | 0.5645 |
| outcome_typeCigarettes:ms_1Yes | 0.594 | 0.1316 | 1.446 | 0.0016 |
| outcome_typeCocaine:ms_1Yes | 1.508 | 0.2679 | 2.118 | 0.1494 |

### Model Diagnostics

Table S1. Model Sample Sizes and Convergence Status

| **Model** | **Sample** | **n** | **Converged** |
| --- | --- | --- | --- |
| M1 | Full | 18,983 | Yes |
| M2a | Full | 16,846 | Yes |
| M2b | Subsample | 10,133 | Yes |
| M3 | Full | 12,270 | Yes |
| M3-sub | Subsample | 10,139 | Yes |
| M4 | Subsample | 9,484 | Yes |
| M5 | Subsample | 9,401 | Yes |
| All models estimated using survey-weighted logistic regression (svyglm, quasibinomial family). Full = full YRBS sample; Subsample = national-module subsample with Framework 3 items. All models converged without warnings. | | | |

Table S2. Variance Inflation Factors for M5 Predictors

| **Predictor** | **VIF** |
| --- | --- |
| Lifetime polysubstance use | 1.19 |
| Risk behavior | 1.09 |
| Bullying victimization | 1.19 |
| Suicidality | 1.27 |
| Sexual victimization | 1.17 |
| Household adversity | 1.24 |
| Community safety adversity | 1.20 |
| VIF computed from the model matrix of the fully adjusted M5 specification. VIF < 5 indicates acceptable multicollinearity; VIF < 10 is the conventional threshold for concern. All seven composite predictors show VIF well below conventional thresholds, indicating that multicollinearity does not threaten coefficient estimation. | |

Table S3. Block-Wise Wald Tests for Incremental Model Improvement

| **Comparison** | **F** | **p** |
| --- | --- | --- |
| M3 vs. M2a (adding F1: polysubstance + risk behavior) | 29.56 | < 0.0001 |
| M4 vs. M3-sub (adding F3: sexual victimization + household adversity) | 14.43 | < 0.0001 |
| M5 vs. M4 (adding community safety adversity) | 23.37 | < 0.0001 |
| Each test evaluates whether the added block of predictors significantly improves model fit beyond the previous specification. All blocks contributed significantly. | | |

Table S4. Composite Summary: Items, Temporal Frame, and Reliability

| **Composite** | **Items** | **Temporal Frame** | **Metric** | **Value** | **95% Boot CI** |
| --- | --- | --- | --- | --- | --- |
| Lifetime polysubstance use | Q31, Q41, Q46 | Lifetime | Alpha | 0.88 | [0.87, 0.88] |
| Risk behavior | Q12, Q17 | Past 30 days/12 months | Spearman-Brown | 0.63 | [0.59, 0.66] |
| Bullying victimization | Q24, Q25 | Past 12 months | Spearman-Brown | 0.87 | [0.86, 0.88] |
| Suicidality | Q27, Q28, Q29 | Past 12 months | Alpha | 0.94 | [0.94, 0.95] |
| Sexual victimization | Q19, Q57 | Lifetime | Spearman-Brown | 0.75 | [0.72, 0.78] |
| Household adversity | Q91, Q100, Q101 | Lifetime/ever | Alpha | 0.81 | [0.79, 0.82] |
| Community safety adversity | Q14, Q15, Q18 | Past 30 days/12 months | Alpha | 0.66 | [0.64, 0.68] |
| All composites coded dichotomously (0 = no items endorsed; 1 = any item endorsed). Boot CI = percentile bootstrap confidence interval (500 resamples). | | | | | |

Table S5. Adjusted Odds Ratios and Average Marginal Effects From Primary Logistic Regression Models Predicting Lifetime NMPOU

| **Predictor** | **M1** | **M2a** | **M3** | **M4** | **M5** | **M5 AME (pp)** |
| --- | --- | --- | --- | --- | --- | --- |
| Female sex | 1.51 (1.33-1.70)*** | 1.22 (1.06-1.40)** | 1.18 (0.99-1.39) | 1.10 (0.92-1.32) | 1.15 (0.96-1.38) | -- |
| Grade 10^th^ | 1.11 (0.91-1.35) | 1.21 (0.97-1.51) | 1.09 (0.84-1.41) | 0.99 (0.74-1.33) | 1.00 (0.75-1.33) | -- |
| Grade 11^th^ | 0.87 (0.71-1.07) | 0.99 (0.80-1.21) | 0.93 (0.74-1.17) | 0.88 (0.68-1.14) | 0.90 (0.68-1.17) | -- |
| Grade 12^th^ | 0.82 (0.64-1.05) | 0.97 (0.75-1.25) | 0.79 (0.59-1.06) | 0.70 (0.52-0.96)* | 0.71 (0.53-0.96)* | -- |
| Black | 1.25 (0.99-1.59) | 1.42 (1.16-1.74)** | 1.30 (1.03-1.64)* | 1.39 (1.09-1.78)* | 1.25 (0.96-1.63) | -- |
| Latine/Multiple Latine | 1.34 (1.08-1.66)** | 1.48 (1.24-1.77)*** | 1.41 (1.19-1.67)*** | 1.42 (1.17-1.73)*** | 1.31 (1.08-1.58)** | -- |
| Multiple Non-Latine | 1.57 (1.11-2.21)* | 1.47 (1.02-2.12)* | 1.17 (0.79-1.72) | 1.11 (0.75-1.63) | 1.03 (0.69-1.53) | -- |
| Other race/ethnicity | 1.03 (0.65-1.65) | 0.98 (0.60-1.59) | 1.18 (0.69-2.02) | 1.27 (0.68-2.35) | 1.23 (0.68-2.22) | -- |
| Lifetime polysubstance use | -- | -- | 2.30 (1.84-2.87)*** | 2.21 (1.74-2.80)*** | 2.09 (1.63-2.66)*** | 6.3 (4.1, 8.5) |
| Risk behavior | -- | -- | 1.85 (1.49-2.30)*** | 1.63 (1.28-2.07)*** | 1.50 (1.18-1.92)** | 4.1 (1.4, 6.8) |
| Bullying victimization | -- | 2.08 (1.78-2.42)*** | 1.88 (1.55-2.28)*** | 1.62 (1.29-2.04)*** | 1.48 (1.18-1.86)** | 3.8 (1.5, 6.0) |
| Suicidality | -- | 3.06 (2.59-3.61)*** | 2.32 (1.94-2.76)*** | 2.00 (1.67-2.38)*** | 1.89 (1.58-2.26)*** | 6.2 (4.2, 8.3) |
| Sexual victimization | -- | -- | -- | 1.49 (1.13-1.95)** | 1.38 (1.03-1.84)* | 3.1 (0.0, 6.3) |
| Household adversity | -- | -- | -- | 1.53 (1.21-1.94)*** | 1.40 (1.12-1.75)** | 3.0 (1.1, 4.9) |
| Community safety adversity | -- | -- | -- | -- | 1.74 (1.39-2.19)*** | 5.2 (3.0, 7.4) |
| N | 18,983 | 16,846 | 12,270 | 9,484 | 9,401 |  |
| AOR columns show adjusted odds ratios (95% confidence intervals). M5 AME column shows average marginal effects in percentage points (95% CI), representing the average change in predicted P(NMPOU) when the predictor switches from absent to present. M1 = demographics only. M2a = demographics + distress variables (bullying, suicidality). M3 = M2a + lifetime polysubstance use + risk behavior. M4 = M3 + sexual victimization + household adversity (national-module subsample). M5 = M4 + community safety adversity (national-module subsample). Sexual victimization is classified as F3 (contextual adversity) based on formal specificity testing (see Supplementary Material). Reference categories: male sex, 9th grade, White race/ethnicity. "--" indicates that the predictor was not included in that model. *p < .05; **p < .01; ***p < .001. | | | | | | |

Table S6. Adjusted Odds Ratios (95% CI) From M5 Specification Across Five Lifetime Substance Outcomes

| **Predictor** | **NMPOU** | **Alcohol** | **Marijuana** | **Cigarettes** | **Cocaine** |
| --- | --- | --- | --- | --- | --- |
| Polysubstance use (LOO) | 2.09 (1.63-2.66)*** | 7.47 (6.03-9.24)*** | 10.25 (7.90-13.30)*** | 15.39 (10.08-23.49)*** | 9.79 (2.88-33.28)*** |
| Risk behavior | 1.50 (1.18-1.92)** | 1.31 (1.02-1.68)* | 1.62 (1.24-2.12)*** | 1.60 (1.24-2.05)*** | 2.58 (1.41-4.73)** |
| Bullying victimization | 1.48 (1.18-1.86)** | 1.06 (0.91-1.25) | 1.07 (0.86-1.33) | 1.04 (0.80-1.34) | 1.12 (0.68-1.85) |
| Suicidality | 1.89 (1.58-2.26)*** | 1.13 (0.97-1.31) | 1.82 (1.50-2.20)*** | 1.61 (1.30-1.99)*** | 2.62 (1.66-4.13)*** |
| Sexual victimization | 1.38 (1.03-1.84)* | 1.05 (0.83-1.32) | 2.35 (1.77-3.13)*** | 1.82 (1.48-2.23)*** | 2.90 (1.46-5.75)** |
| Household adversity | 1.40 (1.12-1.75)** | 1.41 (1.24-1.61)*** | 1.47 (1.19-1.81)*** | 1.84 (1.53-2.23)*** | 0.93 (0.50-1.72) |
| Community safety adversity | 1.74 (1.39-2.19)*** | 1.41 (1.22-1.63)*** | 1.56 (1.29-1.88)*** | 1.12 (0.95-1.33) | 2.47 (1.39-4.37)** |
| Female sex | 1.15 (0.96-1.38) | 1.20 (0.98-1.45) | 1.04 (0.90-1.21) | 0.68 (0.53-0.86)** | 0.44 (0.27-0.73)** |
| N | 9,401 | 9,401 | 9,401 | 9,401 | 9,399 |
| Each column reports adjusted odds ratios (95% CI) from the M5 specification applied to a different lifetime substance outcome. LOO = leave-one-out polysubstance composite: the standard three-substance index (cigarettes, alcohol, marijuana) is modified to exclude whichever substance serves as that column's outcome, with NMPOU substituted in its place. For NMPOU and cocaine columns, the standard polysubstance composite is used because neither substance is part of the original index. All models adjust for sex, grade, and race/ethnicity. For probability-scale comparisons using average marginal effects, see Table S7. *p < .05; **p < .01; ***p < .001. | | | | | |

Table S7. Average Marginal Effects (Probability Scale) by Substance Type

| **Variable** | **NMPOU AME (95% CI)** | **Alcohol AME (95% CI)** | **Marijuana AME (95% CI)** | **Cigarettes AME (95% CI)** | **Cocaine AME (95% CI)** |
| --- | --- | --- | --- | --- | --- |
| Polysubstance use | 0.063 (0.041, 0.085) | 0.438 (0.397, 0.480) | 0.374 (0.341, 0.407) | 0.199 (0.172, 0.227) | 0.021 (0.013, 0.028) |
| Risk behavior | 0.041 (0.014, 0.068) | 0.048 (0.002, 0.095) | 0.073 (0.032, 0.115) | 0.053 (0.023, 0.083) | 0.019 (0.005, 0.033) |
| Bullying victimization | 0.038 (0.015, 0.060) | 0.011 (-0.017, 0.039) | 0.010 (-0.022, 0.042) | 0.004 (-0.023, 0.031) | 0.002 (-0.007, 0.010) |
| Suicidality | 0.062 (0.042, 0.083) | 0.022 (-0.006, 0.050) | 0.092 (0.062, 0.122) | 0.052 (0.028, 0.076) | 0.016 (0.008, 0.024) |
| Sexual victimization | 0.031 (0.000, 0.063) | 0.008 (-0.034, 0.050) | 0.134 (0.089, 0.178) | 0.069 (0.043, 0.095) | 0.022 (0.001, 0.042) |
| Household adversity | 0.030 (0.011, 0.049) | 0.063 (0.040, 0.086) | 0.058 (0.026, 0.089) | 0.063 (0.045, 0.081) | -0.001 (-0.011, 0.009) |
| Community safety adversity | 0.052 (0.030, 0.074) | 0.062 (0.035, 0.090) | 0.067 (0.038, 0.096) | 0.012 (-0.005, 0.030) | 0.014 (0.006, 0.021) |
| AME = average marginal effect on the probability scale (change in P(Y=1) when the binary predictor switches from No to Yes, averaged over all observed covariate values). AMEs are invariant to group-specific residual distributions, addressing the concern that logistic regression coefficients may not be directly comparable across outcome types when residual variance differs (Allison, 1999)^51^. Each substance model uses the M5 specification with leave-one-out polysubstance composites (national-module subsample). Linear probability model sensitivity: distress interaction Wald F = 11.28, p = 0.0001011. Relaxed su_loo constraint sensitivity: distress interaction Wald F = 5.73, p = 0.008521. | | | | | |

Table S8. Empirical Evaluation of Sexual Victimization Framework Classification

| **Test** | **Result** | **Verdict** |
| --- | --- | --- |
| 1. Opioid specificity ratio | Ratio = 0.60 (values < 1.0 indicate general, not opioid-specific, associations) | F3 |
| 2. Substance-specific significance | 4 of 5 substances significant (F2 variables typically show 1-2) | F3 |
| 3. Stacked Wald heterogeneity | p = 0.0194 (significant heterogeneity, but marijuana-dominant, not NMPOU-dominant) | Ambiguous |
| 4. KHB polysubstance mediation | 25.2% mediated (highest of all 6 predictors; F3 mean = 18.1%) | F3 |
| 5. AME profile | Strongest AME for marijuana, not NMPOU (F2 pattern would show NMPOU-dominant) | F3 |
| 6. Tetrachoric correlations | Higher tetrachoric r with F3 items (hh_1, ms_1) than with F2 items (bu_1, sc_1) | F2 |
| Six independent tests evaluated whether sexual victimization (sv_1) behaves empirically like a Framework 2 (Distress Relief) variable or a Framework 3 (Contextual Adversity) variable. Tests used AMEs from drug-specific models (Table S7), the stacked-outcome heterogeneity test (Table S12), and KHB decomposition (Table S11). Overall: 4 tests favor F3, 1 favors F2 (tetrachoric correlations), 1 is ambiguous (Wald heterogeneity is significant but marijuana-dominant rather than NMPOU-dominant). Sexual victimization is a hybrid construct: while it produces emotional distress, the mechanism through which it increases substance use is general trauma-coping rather than the opioid-specific social-pain circuit that characterizes bullying. See scripts/sexual_victimization_F2orF3.Rmd for the complete analysis code. | | |

Table S9. Composite Reliability for Primary Indices

| **Composite** | **Items** | **N** | **Alpha** | **Omega** | **Spearman-Brown** | **95% Boot CI** |
| --- | --- | --- | --- | --- | --- | --- |
| Lifetime polysubstance use | Q31, Q41, Q46 | 15,790 | 0.88 | 0.88 | -- | [0.87, 0.88] |
| Risk behavior (primary) | Q12, Q17 | 17,341 | -- | -- | 0.63 | [0.59, 0.66] |
| Risk behavior (3-item, SM7) | Q10, Q12, Q17 | 16,758 | 0.50 | -- | -- | [0.46, 0.53] |
| Bullying victimization | Q24, Q25 | 19,775 | -- | -- | 0.87 | [0.86, 0.88] |
| Suicidality | Q27, Q28, Q29 | 17,603 | 0.94 | 0.94 | -- | [0.94, 0.95] |
| Sexual victimization | Q19, Q57 | 16,353 | -- | -- | 0.75 | [0.72, 0.78] |
| Household adversity | Q91, Q100, Q101 | 13,024 | 0.81 | 0.81 | -- | [0.79, 0.82] |
| Community safety adversity | Q14, Q15, Q18 | 14,044 | 0.66 | 0.67 | -- | [0.64, 0.68] |
| Alpha = tetrachoric alpha. Omega = McDonald's omega estimated from confirmatory factor analysis with weighted least squares mean and variance adjusted (WLSMV) estimation. Spearman-Brown prophecy formula used for two-item composites. 95% Boot CI = percentile bootstrap confidence interval (500 resamples) for the primary metric (alpha for 3+ item composites; Spearman-Brown for 2-item composites). "--" indicates not applicable or not estimable. | | | | | | |

Table S10. Subsample Validation: M3 Estimated on Full Sample Versus National-Module Subsample

| **Predictor** | **Full Sample AOR** | **Subsample AOR** | **Change (%)** |
| --- | --- | --- | --- |
| Female sex | 1.18 | 1.20 | 1.7 |
| Grade 10th | 1.09 | 1.07 | 1.8 |
| Grade 11th | 0.93 | 0.92 | 1.1 |
| Grade 12th | 0.79 | 0.77 | 2.5 |
| Black | 1.30 | 1.35 | 3.8 |
| Latine/Multiple Latine | 1.41 | 1.43 | 1.4 |
| Multiple Non-Latine | 1.17 | 1.22 | 4.3 |
| Other race/ethnicity | 1.18 | 1.15 | 2.5 |
| Lifetime polysubstance use | 2.30 | 2.30 | 0.0 |
| Risk behavior | 1.85 | 1.80 | 2.7 |
| Bullying victimization | 1.88 | 1.86 | 1.1 |
| Suicidality | 2.32 | 2.24 | 3.4 |
| Full sample N = 12,270. National-module subsample N = 10,139. Change calculated as absolute percentage change in AOR. All changes fall below the pre-specified 15% flag threshold, confirming analytic sample comparability. Maximum AOR change = 4.3%. | | | |

Table S11. KHB Mediation Decomposition: Proportion of Predictor-NMPOU Associations Mediated by Lifetime Polysubstance Involvement

| **Variable** | **Total AOR** | **Direct AOR** | **Indirect (log-odds)** | **% Mediated** | **95% CI** |
| --- | --- | --- | --- | --- | --- |
| Bullying victimization | 1.88 | 1.77 | 0.066 | 10.3 | 3.2 to 17.5 |
| Suicidality | 2.52 | 2.18 | 0.142 | 15.3 | 9.6 to 21.1 |
| Sexual victimization | 1.77 | 1.53 | 0.144 | 25.2 | 11.4 to 39.0 |
| Risk behavior | 1.99 | 1.76 | 0.127 | 18.4 | 10.2 to 26.5 |
| Household adversity | 1.54 | 1.4 | 0.094 | 21.8 | 5.6 to 38.0 |
| Community safety adversity | 1.91 | 1.74 | 0.092 | 14.2 | 5.5 to 22.9 |
| KHB = Karlson-Holm-Breen decomposition. Total effect = association before adjusting for polysubstance involvement (from model with demographics + all other predictors, replacing su_life with its residuals). Direct effect = association after adjusting for polysubstance involvement (from model with su_life included). Indirect effect = total minus direct on the log-odds scale. Proportion mediated = indirect / total on the log-odds scale. Cross-sectional KHB results are interpreted as accounting decompositions, not causal mediation pathways. Bullying, suicidality, sexual victimization, and risk behavior: N = 11,878 complete cases (full sample). (a) Household adversity and community safety adversity: N = 9,401 complete cases (national-module subsample). | | | | | |

Table S12. Three-Substance Confirmatory Model: Key Interaction Coefficients

| **Interaction** | **Beta** | **SE** | **p** |
| --- | --- | --- | --- |
| Bullying x Alcohol | -0.37 | 0.16 | 0.0261 |
| Bullying x Marijuana | -0.43 | 0.17 | 0.0178 |
| Suicidality x Alcohol | -0.43 | 0.13 | 0.00221 |
| Suicidality x Marijuana | 0.00 | 0.14 | 0.996 |
| Sexual victimization x Alcohol | -0.11 | 0.22 | 0.617 |
| Sexual victimization x Marijuana | 0.65 | 0.20 | 0.00314 |
| Risk behavior x Alcohol | -0.20 | 0.21 | 0.36 |
| Risk behavior x Marijuana | 0.04 | 0.19 | 0.829 |
| Household adversity x Alcohol | 0.15 | 0.17 | 0.38 |
| Household adversity x Marijuana | 0.17 | 0.20 | 0.405 |
| Community safety x Alcohol | -0.28 | 0.15 | 0.0842 |
| Community safety x Marijuana | -0.10 | 0.17 | 0.548 |
| NMPOU is the reference outcome. Negative interaction coefficients indicate that the variable's association is stronger for NMPOU than for the comparison substance. Positive coefficients indicate the reverse. Joint Wald test of F2 distress interactions (bullying + suicidality, 4 terms): F = 8.24, p = 0.0001765. Sexual victimization interactions are included in the table for completeness but tested separately as an F3 variable. | | | |

Table S13. Attenuation of Predictor Associations Across Model Sequence

| **Variable** | **M2a AOR** | **M3 AOR** | **M3-sub AOR** | **M4 AOR** | **M5 AOR** | **M2a to M3 (%)** | **M3-sub to M5 (%)** |
| --- | --- | --- | --- | --- | --- | --- | --- |
| Lifetime polysubstance use | -- | 2.30 | 2.30 | 2.21 | 2.09 | -- | 11.8% |
| Risk behavior | -- | 1.85 | 1.80 | 1.63 | 1.50 | -- | 30.7% |
| Bullying victimization | 2.08 | 1.88 | 1.86 | 1.62 | 1.48 | 13.6% | 36.5% |
| Suicidality | 3.06 | 2.32 | 2.24 | 2.00 | 1.89 | 24.8% | 21.1% |
| Sexual victimization | -- | -- | -- | 1.49 | 1.38 | -- | -- |
| Household adversity | -- | -- | -- | 1.53 | 1.40 | -- | -- |
| Community safety adversity | -- | -- | -- | -- | 1.74 | -- | -- |
| Attenuation calculated on the log-odds scale as (beta_earlier - beta_later) / beta_earlier x 100, where beta is the raw regression coefficient. This avoids distortion from rounding when using exponentiated AORs. M2a = demographics + distress (full sample). M3 = M2a + polysubstance use + risk behavior (full sample). M3-sub = M3 estimated on national-module subsample. M4 = M3-sub + household adversity. M5 = M4 + community safety adversity. All models adjust for sex, grade, and race/ethnicity. | | | | | | | |

Table S14. Survey-Weighted Characteristics of NMPOU-Positive vs. NMPOU-Negative Adolescents

| **Variable** | **NMPOU+ (%)** | **NMPOU- (%)** | **p-value** |
| --- | --- | --- | --- |
| Female sex | 58.0 | 47.0 | < .001 |
| Race/Ethnicity |  |  | 0.007 |
| White | 41.6 | 48.9 | 0.007 |
| Black | 14.2 | 13.1 |  |
| Latine/Multiple Latine | 31.9 | 27.0 |  |
| Multiple Non-Latine | 7.8 | 5.9 |  |
| Other | 4.5 | 5.1 |  |
| Polysubstance use | 73.6 | 49.2 | < .001 |
| Risk behavior | 20.8 | 9.1 | < .001 |
| Bullying victimization | 46.4 | 22.3 | < .001 |
| Suicidality | 51.5 | 20.8 | < .001 |
| Sexual victimization | 22.6 | 8.1 | < .001 |
| Household adversity | 66.7 | 40.7 | < .001 |
| Community safety adversity | 59.1 | 30.5 | < .001 |
| Percentages are survey-weighted. p-values from design-adjusted Rao-Scott chi-square tests. Full-sample predictors: polysubstance use, risk behavior, bullying, suicidality, sexual victimization. National-module subsample predictors: household adversity, community safety adversity. | | | |

**Interpretation.** Table S14 compares survey-weighted prevalence of demographic and composite predictor characteristics between NMPOU-positive and NMPOU-negative adolescents. All seven composite predictors showed significantly higher endorsement among NMPOU-positive adolescents, consistent with the multivariate associations in Table S5. These bivariate differences illustrate the elevated adversity burden among adolescents who report NMPOU and underscore the importance of the multivariate adjustment in Models M1-M5 to distinguish independent, opioid-specific associations from general comorbidity.

## Figure S1. Framework Endorsement by Sex

Figure S1 displays three-framework Euler diagrams stratified by sex. Among female NMPOU-positive adolescents, the three-way F1-F2-F3 intersection was consistently larger than among males, reflecting higher rates of distress and adversity co-endorsement. Among male NMPOU-positive adolescents, F1 (risk-taking) occupied a relatively larger proportion. These sex-stratified patterns are consistent with differential developmental pathways to substance use: females are more likely to follow internalizing pathways (distress, adversity) whereas males more often follow externalizing pathways (risk-taking, sensation-seeking).^38,59^

**Sensitivity Analyses Overview.** Eleven pre-specified sensitivity analyses tested whether the primary findings held under alternative measurement decisions. Each analysis changed one element of the primary model and re-estimated the key parameters. SM1 replaced suicidality with persistent sadness; SM2 added dating violence; SM3 and SM5 tested different operationalizations of sexual victimization (forced intercourse, three-level debut); SM4 expanded household adversity to include parental incarceration; SM6 substituted past-30-day polysubstance involvement for lifetime use; SM7 modified the risk behavior composite; SM8 restricted the sample to national-module respondents; SM9 used past-30-day NMPOU as the outcome; SM10 tested an alternative polysubstance composite including cocaine; and SM11 entered weapon carrying (Q12) and physical fighting (Q17) as separate predictors in place of the 2-item risk behavior composite. The table below summarizes each analysis, the variable tested, the resulting AOR and 95% CI, and the sample size. Across all eleven analyses, the primary findings remained consistent: polysubstance use was the strongest overall predictor, and bullying retained its opioid-specific association.

Table S15. Sensitivity Analyses Summary (SM1-SM11)

| **Model** | **Modification** | **Variable Tested** | **AOR** | **95% CI** | **N** |
| --- | --- | --- | --- | --- | --- |
| SM1 | Persistent sadness replaces suicidality | Persistent sadness (Q26) | 1.88 | 1.53-2.32 | 9,680 |
| SM2 | Dating violence added (daters only) | Dating violence (Q21, Q22) | 1.06 | 0.77-1.47 | 9,217 |
| SM3 | Forced sexual intercourse added to M5 | Forced sexual intercourse (Q19) | 1.31 | 0.98-1.76 | 9,495 |
| SM4 | Expanded household adversity (+ Q102) | Expanded household adversity (Q91, Q100, Q101, Q102) | 1.44 | 1.13-1.85 | 9,564 |
| SM5a | Early debut added (age 12 or younger) | Early debut (12 or younger) | 1.89 | 1.15-3.11 | 9,506 |
| SM5b | Early debut added (age 13 or older) | Early debut (13 or older) | 1.28 | 1.04-1.57 |  |
| SM6 | Past-30-day polysubstance (M3 spec) | Past-30-day polysubstance use (Q33, Q42, Q48) | 1.94 | 1.59-2.36 | 14,252 |
| SM7 | Three-item risk behavior (M3 spec) | Risk behavior, 3-item (Q10, Q12, Q17) | 1.22 | 1.00-1.50 | 12,103 |
| SM8 | Restricted to national-module respondents | Bullying victimization (Q24, Q25) | 1.86 | 1.51-2.30 | 10,139 |
| SM9 | Past-30-day NMPOU as outcome (Q92) | Suicidality (Q27, Q28, Q29) | 2.19 | 1.64-2.93 | 9,403 |
| SM10 | Alternative polysubstance composite including cocaine | Bullying victimization (Q24, Q25) | 1.50 | 1.20-1.87 | 9,380 |
| SM11a | Weapon carrying and physical fighting as separate predictors | Weapon carrying (Q12) | 1.60 | 1.14-2.25 | 9,401 |
| SM11b | Weapon carrying and physical fighting as separate predictors | Physical fighting (Q17) | 1.31 | 0.91-1.89 |  |
| All models adjust for demographics (sex, grade, race/ethnicity). SM1 through SM5 use lifetime NMPOU (Q49) as the outcome and follow the M5 specification unless otherwise noted. SM6 and SM7 modify the M3 specification (full sample, no F3 variables). SM8 restricted the sample to national-module respondents (M3 specification). SM9 used past-30-day NMPOU (Q92) as the outcome. SM10 tested an alternative polysubstance composite including cocaine (cigarettes, alcohol, marijuana, and cocaine). SM11 entered weapon carrying (Q12) and physical fighting (Q17) as separate predictors in place of the 2-item risk behavior composite (rb_1). SM3 suicidality attenuation after adding forced sexual intercourse: 4.4%. The 2-item risk behavior composite (SM7) produced a significant association, whereas the 3-item version (adding Q10: riding with drunk driver) did not, reflecting measurement sensitivity to composite specification. AOR = adjusted odds ratio; CI = confidence interval. | | | | | |

**Interpretation.** The eleven sensitivity analyses consistently supported the primary findings. First, when persistent sadness replaced suicidality (SM1), the association with NMPOU remained significant and comparable in magnitude, indicating that the distress pathway is not driven by one specific operationalization of internalizing distress. Second, adding dating violence (SM2) or forced sexual intercourse alone (SM3) did not meaningfully alter the existing predictor associations, confirming that the primary model adequately captures victimization-related risk. Third, expanding household adversity to include parental incarceration (SM4) and testing a three-level sexual debut variable (SM5) produced similar results, suggesting that the primary composites are robust to item expansion. Fourth, substituting past-30-day polysubstance use for lifetime use (SM6) and modifying the risk behavior composite to include riding with a drunk driver (SM7) yielded consistent patterns, though the risk behavior finding was sensitive to composite specification (SM7), which is noted as a limitation. Fifth, restricting the sample to national-module respondents (SM8) confirmed that the two-sample strategy did not bias the primary results. Sixth, using past-30-day NMPOU as an alternative outcome (SM9) replicated the overall pattern. Seventh, including cocaine in the polysubstance composite (SM10) did not alter the conclusions. Eighth, entering weapon carrying and physical fighting as separate predictors (SM11) rather than a composite allowed examination of each item’s independent association with NMPOU. Across all analyses, polysubstance use remained the strongest predictor and bullying retained its opioid-specific association, reinforcing the robustness of the primary findings.
